# Sensitivity evaluation and multi-omics analysis of *Bursaphelenchus xylophilus* populations to emamectin benzoate in Shandong province field

**DOI:** 10.3389/fpls.2026.1894612

**Published:** 2026-10-15

**Authors:** Kunyang Ju, Zhifan Ma, Shuhan Guo, Yuke Wang, Shengnan Dong, Gengwu Sun, Yilin Wang, Jinzhu Zheng, Huixiang Liu

**Affiliations:** 1 College of Plant Protection, Shandong Agricultural University, Tai’an, China; 2 Shandong Research Center for Forestry Harmful Biological Control Engineering and Technology, Shandong Agricultural University, Tai’an, China; 3 College of Forestry, Shandong Agricultural University, Tai’an, China; 4 College of Plant Protection, Nanjing Agricultural University, Nanjing, China; 5 Forestry Development Center of Wendeng District, Weihai, China; 6 Zhifu District Forest Fire Prevention and Protection Support Service Center, Yantai, China; 7 Culai Forest Farm of Shandong Province, Tai’an, China

**Keywords:** Bursaphelenchus xylophilus, emamectin benzoate, nematicide, resistance monitoring, RNA interference

## Abstract

Pine trees provide substantial ecological and economic benefits worldwide, including water conservation, timber production, and carbon sequestration. However, *Bursaphelenchus xylophilus*, as the pathogen of pine wilt disease (PWD), results in extensive pine mortality annually, posing a serious threat to global pine resources and environmental security. Currently, chemical control remains the primary strategy for managing PWD. In Shandong Province, emamectin benzoate (EB) is the main nematicide for trunk injection. Nevertheless, long-term use of a single compound carries a high risk of resistance development. In this study, the sensitivity of 15 *B. xylophilus* strains from different regions of Shandong Province to EB was evaluated. The highest and lowest LC_50_ values were observed in the PL (320.99 μg•mL^-1^) and CD strains (114.63 μg•mL^-1^), respectively, yielding a resistance ratio (PL vs CD) of 2.8 < 5, indicating that EB resistance may not have developed in the *B. xylophilus* populations in Shandong. After EB treatment, the PL strain exhibited significantly higher detoxification enzyme activities and lower accumulation of toxic aldehydes than the CD strain. Multi-omics analysis was further performed to explore the molecular basis for the different sensitivity, leading to the identification of the glycolysis/gluconeogenesis pathway and *BX03G0588* as the potentially responsive pathway and candidate gene, respectively. Following ds0588 treatment, the sensitivity of the PL strain to EB was significantly enhanced, confirming the function of *BX03G0588* relating to the sensitivity of nematode. This study provided new insights into the development of resistance and sustainable management strategies against PWD.

## 1 Introduction

Pines are an essential component of forest ecosystems, playing critical roles in water conservation, sand fixation and dust retention (Susaeta et al., 2023; Delgado-Rodriguez et al., 2025; Gupta et al., 2023). While conserving ecosystems, pine trees also possess extremely high economic value, with industrial output dominated by pine timber and resin accounting for an important proportion (Mo et al., 2023; Lai et al., 2023). However, the outbreak of pine wilt disease (PWD) has caused widespread pine mortality, resulting in global economic losses each year (Liang et al., 2018). Since its first detection in China in 1982, it causes an average annual economic loss of 717 million RMB and the death of more than 14 million pine trees (Zhao et al., 2020).

*Bursaphelenchus xylophilus* (Steiner & Buhrer) Nickle is the pathogenic agent of pine wilt disease, which can trigger massive pine death within a short period (Feng et al., 2022). As a plant-parasitic nematode, *B. xylophilus* is primarily transmitted by vector insects of the *Cerambycidae* (Mitrea-Calin et al., 2020). In Shandong Province of China, the nematode has rapidly spread to most regions relying on *Monochamus alternatus*. When beetles feed on pine branches, *B. xylophilus* escape from the respiratory tract of the beetles, invade the xylem vessels of pine trees and cause vessel blockage, thereby impeding water transport (Akami and Fukuda, 2025; Koo et al., 2013). Relying on this transmission route, *B. xylophilus* has spread widely across Asia and Europe, inflicting devastating damage on local pine industrial chains (Cha et al., 2019).

In the early infection stage, external symptoms are inconspicuous, nematodes are invisible to the naked eye, and only a small number of pine needles exhibit slight yellowing and reduce chlorophyll content (Xu et al., 2011). When the pine needles turn yellow and resin secretion reduces, the infected pine trees are incurable. At present, a comprehensive early monitoring such as infrared spectroscopy and management system for PWD has been initially established (Pan et al., 2018). Chemical control remains the dominant approach in these strategies. The combined application of aerial spraying and trunk injection can effectively block the migration path of *Monochamus alternatus* and suppress nematodes’ activity in pine trees. In Shandong Province, emamectin benzoate (EB) is the major nematicide applied for *B. xylophilus* management with excellent stability and a long persistent period in pine tissues (Zhang et al., 2023). As a 16-membered macrocyclic lactone compound derived from the amination modification of avermectin, its target is the glutamate-gated chloride channel (GluCl) (Huang et al., 2024). Meanwhile, it sustains the opening of ion channels, leading to massive influx of chloride ions and subsequent cellular hyperpolarization, which causes rapid paralysis and death of nematodes in a short time (Lee et al., 2023). With the escalating harm of PWD, the application dosage of EB has gradually increased. Especially for trunk injection as a labor-intensive management strategy, increasing the application dosage can maintain the persistent efficacy and effective concentration of EB in pine trees, thereby reducing labor consumption (Lee et al., 2020; Zhang et al., 2023). Nevertheless, long-term application of a single nematicide can induce nematode resistance (Wang et al., 2026). The pest resistance to EB has been reported. Target gene mutations and overexpression of cytochrome P450 family genes conferred resistance to EB in *Frankliniella occidentalis* and *Spodoptera exigua* (Zeng et al., 2026; Gao et al., 2022). At present, studies on the effects of EB against *B. xylophilus* mainly focus on two aspects. Potential target sites of EB are identified based on the expression levels of genes related to the GluCl subunit and molecular docking analysis (Huang et al., 2024; Lu et al., 2020). In addition, the nematicidal efficacy of EB has been improved via formulation optimization, including nanoformulation, emulsifiable concentrate preparation and compounding with other nematicide (Teng et al., 2025; Lin et al., 2024; Li et al., 2024; Zhang et al., 2026). However, persistent EB application, the distinct transmission pattern, and the parasitic life traits may lead to the development of resistance of *B. xylophilus* in field populations and arouse our strong concerns.

Therefore, this study determined the sensitivity of *B. xylophilus* strains from different regions of Shandong Province to EB. Combined with multi-omics analysis, we further explored the formation possibility and internal molecular mechanism of EB resistance in Shandong Province and provide sustainable management strategies for the scientific application of nematicides in field production.

## 2 Materials and methods

### 2.1 Chemicals and nematodes

Emamectin benzoate (95% purity) was obtained from Syngenta Crop Protection Co., Ltd. (China). Trizol reagent was purchased from Invitrogen (Thermo Fisher Scientific Inc., Waltham, MA, USA). The T7 RNAi Transcription Kit was supplied by Vazyme Co., Ltd. (China). Enzyme activity assay kits were purchased from Beijing Solarbio Science & Technology Co., Ltd. (China). Nematodes were isolated from the infected wood of different regions of Shandong Province, China using the Baermann funnel method (**Table 1**). All isolates were first identified morphologically, and then confirmed by PCR. The specific primer sequences of *B. xylophilus* was provided in **Table 2**. Different strains were subcultured once on *Botrytis cinerea*-covered potato dextrose agar (PDA) for subsequent toxicity assay and avoiding interference caused by laboratory adaptation.

**Table 1:** Sampling Regions and geographic coordinates.

Population	Region	Longitude/latitude
CD	Changdao	120.74/37.93
PL	Penglai	120.67/37.78
FS	Fushan	121.28/37.38
ZF	Zhifu	121.36/37.44
QX	Qixia	121.23/37.28
ls	Lanshan	119.29/35.20
LS	Laishan	121.42/37.37
K1	Muping	121.72/37.44
CS	Rongcheng	122.70/37.39
WD	Wendeng	122.19/37.26
HC	Huancui	122.03/37.39
RS	Rushan	121.60/36.91
CY	Chengyang	120.46/36.21
HD	Huangdao	119.94/35.92
JM	Jimo	120.58/36.33

**Table 2:** Primer sequences for PCR and RT-qPCR.

Genes	Primer sequence	
	Forward primers (5’-3’)	Reverse primers (5’-3’)
PX1	GATGATGCGATTGGTGACT	CAGTGCGTAGAAGATATTGGT
Actin	CGCAAATACTCCGTCTGGATTGG	TTCGTCGTACTCTTGCTTGGAGA
*BX03G0588*	AAAGAATCGGAAGTCCGACC	CAAAGTTGCACATTCCGTGG
*BX06G0633*	AGTCACAGTCTACAAAGCTCTG	CCCGAGATTTCGCATTTTTGC

### 2.2 Toxicity assay

With the 375 μg•mL^-1^ acetone solution as the control, approximately 100 nematodes were exposed to 1 mL of EB solution at concentrations including 75, 150, 225, 300 and 375 μg•mL^-1^ for 24 h. All treatments were conducted at 25 °C in 24-well plates with three biological replicates. Nematode mortality was assessed under a stereomicroscope using a picking needle (Lee et al., 2023). Nematodes were judged to be dead when they showed no movement after being prodded. Corrected mortality rate (%) was calculated as:


$$\text{Corrected mortality }(\%)=\frac{{\text{Mortality}}_{\text{Treatment}}\;–{\text{ Mortality}}_{\text{Control}}}{1\;–{\text{ Mortality}}_{\text{Control}}}\times 100$$

In the absence of a specific criterion for sensitivity testing and limited sample size for baseline, Insecticide Resistance Action Committee (IRAC) diagnostic criteria for arthropods were used to compare the LC_50_ values among different strains.

### 2.3 Effects of EB on biochemical parameters

Approximately 0.1g nematodes from PL and CD strains were collected and soaked in 39 μg•mL^-1^ EB solution (LC_20_ of CD, 1 mL) for 24 h at 25 °C in the dark, with sterile water as control and three biological replicates. Treatment with sublethal concentrations of EB can effectively reflect changes in the biochemical parameters of nematodes. Then the treated nematodes were wash three times using sterile water and immediately frozen in liquid nitrogen to measure the activities of glutathione-S-transferase (GST), peroxidase (POD), superoxide dismutase (SOD) and malondialdehyde (MDA) levels. Meanwhile, biomass was normalized uniformly by measuring total protein content in every samples.

### 2.4 Screening of the potential pathway relating the sensitivity of nematodes

For transcriptomics and metabolomics (four and six replicates, respectively), nematodes were soaked in 39 μg•mL^-1^EB (1 mL) for 24 h with sterile water as the control.

Total RNA was isolated from nematodes with Trizol reagent, adhering to the manufacturer’s standard protocols. The integral and pure RNA were evaluated via an Agilent 2100 Bioanalyzer and Nanodrop 1000 spectrophotometer. Qualified RNA samples with an RNA integrity number (RIN) above 7.0 were retained for subsequent library construction. mRNA was enriched through two rounds of purification and then chemically fragmented into short reads after divalent cation treatment. The fragmented RNA was reverse-transcribed to generate cDNA. Then, cDNA products were processed by end repair, A-tailing, and adapter ligation. Target fragments were further purified and size-selected with magnetic beads. After UDG digestion and PCR amplification, the cDNA libraries were constructed and subjected to high-throughput sequencing on the Illumina Novaseq™ 6000 platform by LC-Bio Technology Co., Ltd. (Hangzhou, China). The DESeq2 package was applied to screen differentially expressed genes (DEGs), with the threshold set at *p* < 0.05 and fold change > 2 or < 0.5. Both Gene Ontology (GO) functional annotation and Kyoto Encyclopedia of Genes and Genomes (KEGG) pathway enrichment analyses were performed based on the hypergeometric distribution algorithm.

For metabolomic analysis, thirty milligrams of each sample were accurately weighed into a 2 mL centrifuge tube, and a methanol/acetonitrile solution (1:1, v/v) was added thereto. The mixture was vor-texed for 30 s and then homogenized for 30 min, followed by incubation at − 20 °C for 10 min. After centrifugation at 18, 400 × g and 4 °C for 20 min, 200 μL of the supernatant was collected to prepare samples for LC-MS analysis (Ma et al., 2022). Quality control (QC) samples were prepared by pooling equal volumes of extracts from all samples, with the volume of QC samples consistent with that of the test samples. LC-MS analysis was performed on a high-resolution LC-MS platform from Thermo. Chromatographic conditions: ACQUITY UPLC HSS T3 column (100 Å, 1.8 μm, 2.1 mm × 100 mm), the mobile phases consisted of mobile phase A (0.1% formic acid in water, v/v) and mobile phase B (0.1% formic acid in acetonitrile, v/v). The gradient elution program was set as follows: 0 – 1.0 min, mobile phase B was maintained at 5%; 1.0–4.7 min, mobile phase B was linearly increased to 95%; 4.7 – 6.0 min, mobile phase B was maintained at 95%; 6.0 – 6.1 min, mobile phase B was rapidly decreased to 5%; 6.1 – 8.5 min, mobile phase B was maintained at 5% to equilibrate the column, with a total analysis cycle of 8.5 min. The flow rate was 0.4 mL•min^-1^, column temperature was 40 °C, and injection volume was 2 μL. Mass spectrometric conditions: Thermo Orbitrap Exploris 120 mass spectrometer controlled by Xcaliburv4.3, with da-ta-dependent acquisition (DDA) in positive/negative ion modes. HESI source parameters: spray voltage 3.5 kV − 3.0 kV, capillary temperature 320 °C; MS1 resolution 60, 000 (scan range 70–1000 m/z), MS2 resolution 15, 000 (HCD collision energy 30%). Raw data were processed using MS-DIAL v4.9 for peak extraction, alignment, and filtering, as well as metabolite annotation. Missing values were imputed via the Gap filling algorithm followed by data normalization. Metabolite identification was achieved by matching the accurate mass and MS2 spectra against the PSNGM database. Principal component analysis (PCA) and orthogonal partial least squares-discriminant analysis (OPLS-DA) were performed using SIMCA v16.0 on all identified metabolites to investigate global metabolic variations, inter-group differences and intra-group sample discrepancies. Based on OPLS-DA results, metabolites with variable importance in projection (VIP) ≥ 1 and *p* ≤ 0.05 (calculated via the Benjamini-Hochberg method for multiple test-ing correction) were selected as differential metabolites.

The correlation between pathways and genes was constructed for deep transcriptomics analysis using R. Initially, the KEGG enrichment result file was preprocessed to eliminate invalid data. Pairwise analysis was performed on all enriched pathways to quantify the number of common genes between each pathway pair and establish a pathway interaction network. The degree, defined as the count of effective interactions (shared genes > 0) among distinct pathways, was adopted as the key evaluation index. Pathways were sorted according to degree in descending order. Combined with the top five pathways and the results from integrated transcriptomic and metabolomic analysis, glycolysis/gluconeogenesis pathway was selected as potentially responsive pathway.

Gene expression levels of *BX03G0588* and *BX06G0633* in PL strain were analyzed by RT-qPCR, compared with CD strain. The primer sequences were provided in **Table 2**.

### 2.5 Effects of dsRNA on the sensitivity of *B. xylophilus* to EB

The T7 RNAi Transcription Kit was employed for the synthesis of dsRNAs including GFP, *BX03G0588*, and *BX06G0633*. The primer sequences were provided in **Table 3**. Approximately 10, 000 nematodes were exposed to 800 ng•μL^-1^ dsRNA and then cultured under dark conditions at 25 °C for 24 h (Nian et al., 2026). Then, nematodes were washed three times to eliminate residual dsRNA using sterile water. Gene expression levels of *BX03G0588* and *BX06G0633* in PL strain were analyzed by RT-qPCR, with dsGFP as a negative control. To assess the significance of the two genes, the corrected mortality rate of PL strain, soaked in 39 μg•mL^-1^ EB alone and a mixture (800 ng•μL^-1^ dsRNA + 39 μg•mL^-1^ EB, 1mL) for 12 h, was calculated with three biological replicates and sterile water as the control. With the same method in Section 2.2, the PL strain was exposed in a mixture (800 ng•μL^-1^ ds0588 + EB, 1mL) and then the LC_50_ value was measured.

**Table 3:** Primer sequences for dsRNA synthesis.

Genes	Primer sequence	
	Forward primers (5’-3’)	Reverse primers (5’-3’)
T7GFP	TAATACGACTCACTATAGGG ATGGTGAGCAAGGGCGAGGA	TAATACGACTCACTATAGGG TTACTTGTACAGCTCGTCCATGC
dsGFP	ATGGTGAGCAAGGGCGAGGA	TTACTTGTACAGCTCGTCCATGC
T7*BX03G0588*	TAATACGACTCACTATAGGG AACCAACGTCACCAACTTCA	TAATACGACTCACTATAGGG CCGTTCCTCGTGGGCGAAT
ds0588	AACCAACGTCACCAACTTCA	CCGTTCCTCGTGGGCGAAT
T7*BX06G0633*	TAATACGACTCACTATAGGG CCATTCCCGACCAAGCCCTT	TAATACGACTCACTATAGGG GAGCAATTGAACCGCTGACA
ds0633	CCATTCCCGACCAAGCCCTT	GAGCAATTGAACCGCTGACA

### 2.6 Statistical analysis

Data analyses were performed using SPSS 22.0 (IBM Inc., Armonk, NY, USA). Figures were generated using GraphPad Prism 9.5.0 (GraphPad Software Inc., San Diego, CA, USA). Transcriptome correlation analysis was conducted using R 4.2.1 (R Core Team, Vienna, Austria). Network construction for multi-omics integrated analysis was performed with Cytoscape 3.9.1 (Cytoscape Consortium, San Diego, CA, USA).

## 3 Result

### 3.1 Toxicity assay

The LC_50_ values of 15 *B. xylophilus* strains from different regions of Shandong Province were determined to compare the sensitivity to EB. The results showed that the PL strain had the highest LC_50_ value (320.99 μg•mL^-1^), while the CD strain exhibited the lowest LC_50_ value (114.63 μg•mL^-1^), and the resistance ratio (RR) was 2.8. Meanwhile, the sublethal concentration of CD stain was 39.41 μg•mL^-1^. The LC_50_ values of the other strains were presented in **Table 4**.

**Table 4:** The toxicity of emamectin benzoateon to different strains in Shandong Province.

Strain	LC_50_(μg•mL^-1^)	R^2^	Toxicity regression equation
CD	114.63	0.9916	Y=1.2627 + 1.8148X
PL	320.99	0.9777	Y=1.6908 + 1.5789X
FS	158.37	0.9296	Y=-1.1513 + 3.4827X
ZF	199.74	0.8869	Y=2.2757 + 1.3629X
QX	150.36	0.9597	Y=1.7395 + 1.5672X
ls	194.08	0.8526	Y=2.0139 + 1.5131X
LS	148.97	0.9731	Y=0.6934 + 2.0570X
K1	237.01	0.9794	Y=2.4702 + 1.4530X
CS	230.53	0.8966	Y=2.6909 + 0.9773X
WD	297.71	0.9180	Y=1.4287 + 1.4437X
HC	255.73	0.9323	Y=3.1633 + 0.7628X
RS	146.93	0.8266	Y=2.0865 + 1.3448X
CY	191.44	0.9112	Y=0.5847 + 2.2514X
HD	286.48	0.9472	Y=2.7600 + 0.9117X
JM	156.11	0.9723	Y=-2.7863 + 4.3769X
PL (EB+ds0588)	162.56	0.9751	Y=-1.2844 + 2.823X

### 3.2 Effects of EB on biochemical parameters of *B. xylophilus*

Under the EB treatment, the MDA level (0.87 nmol•mg^-1^ prot) of the PL strain was significantly lower than that of the CD strain (**Figure 1A**). The activities of SOD, GST and POD in the PL strain were significantly higher than those in the CD, with the values of 2.92 U•mg^-1^ prot, 5.672 nmol•min^-1^•mg^-1^ prot and 12.07 U•mg^-1^ prot, respectively (**Figure 1B-D**).

**Figure 1:**
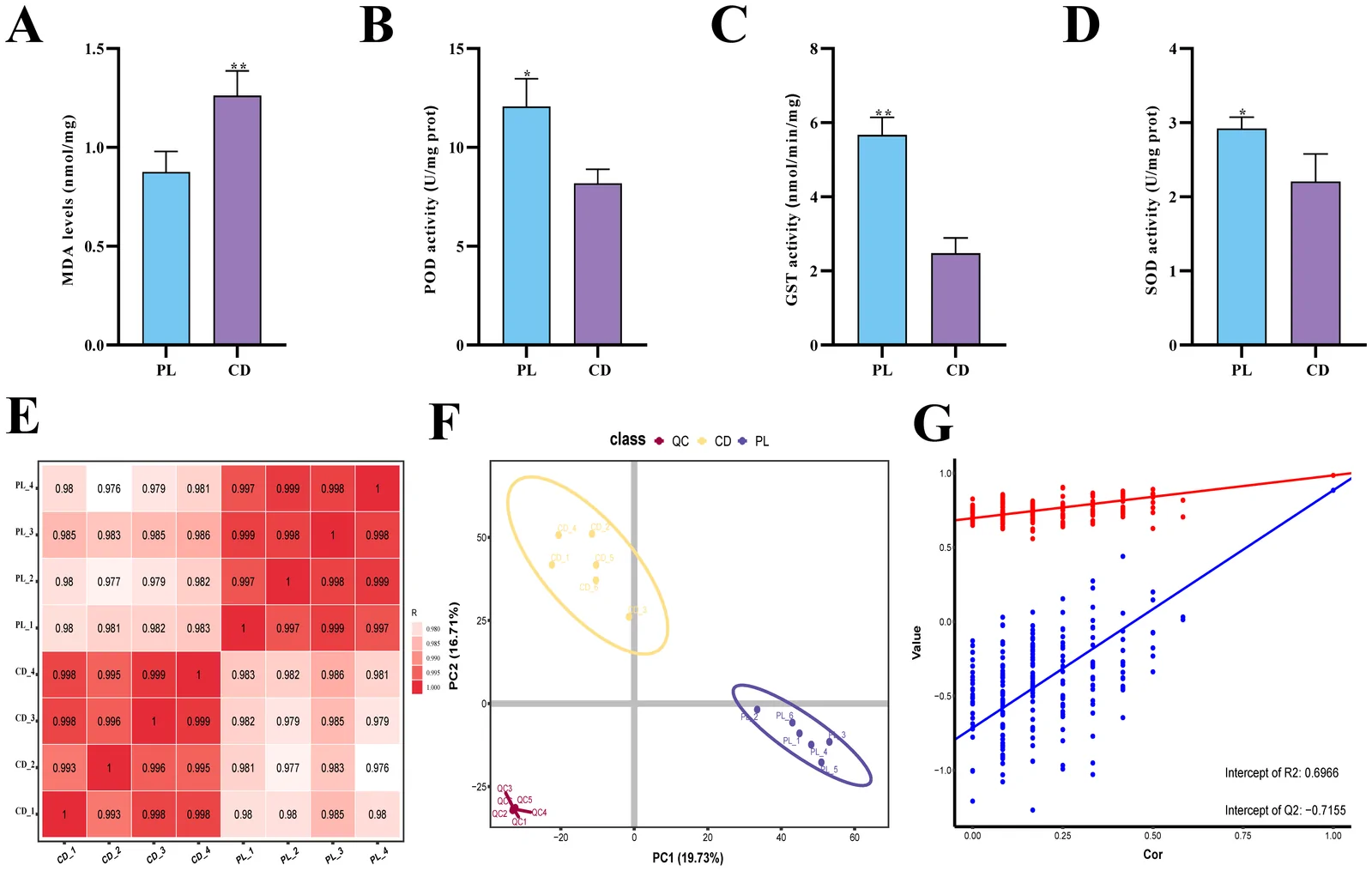
Effects of emamectin benzoateon on PL and FS strains. **(A)** The MDA levels in PL and FS strains (n=3). **(B-D)** The activities of POD, GST, and SOD in PL and FS stains (n=3). **(E)** Transcriptomic pearson correlation between samples after 24-hour EB treatment. **(F, G)** Metabolomic PLS-DA score and permutation test plot after 24-hour EB treatment. Differences between the two groups were analyzed using t-test. *p < 0.05 and **p < 0.01.

### 3.3 Screening of the potential pathway relating to the sensitivity of nematode

Sequencing generated 45.03 GB of clean data, with a Q30 base percentage of 98.54% and an average of 11.23 GB per sample, confirming that the sequencing quality was sufficient for further analysis (**Table 5**). PL and CD strains both showed very high within-group consistency indicating experimental reproducibility and clear molecular differences between the two strains (**Figure 1E**).

**Table 5:** Statistical analysis of transcriptomic data after EB treatment.

Sample	Raw bases	Valid bases	Q30%
PL-1	5.87G	5.78G	98.52
PL-2	5.58G	5.48G	98.49
PL-3	5.37G	5.29G	98.53
PL-4	5.99G	5.89G	98.61
CD-1	5.37G	5.23G	98.49
CD-2	5.80G	5.67G	98.54
CD-3	5.66G	5.55G	98.59
CD-4	6.24G	6.14G	98.57

In the PL group, we identified differentially expressed genes (DEGs), of which 457 were upregulated and 72 were downregulated compared with the CD group. GO annotations primarily related to the obsolete oxidation-reduction process, membrane, and hydrolase activity (**Figure 2A**). KEGG pathway enrichment revealed significant involvement of lysosome, drug metabolism-cytochrome P450, and metabolism of xenobiotics by cytochrome P450 (**Figure 2B**).

**Figure 2:**
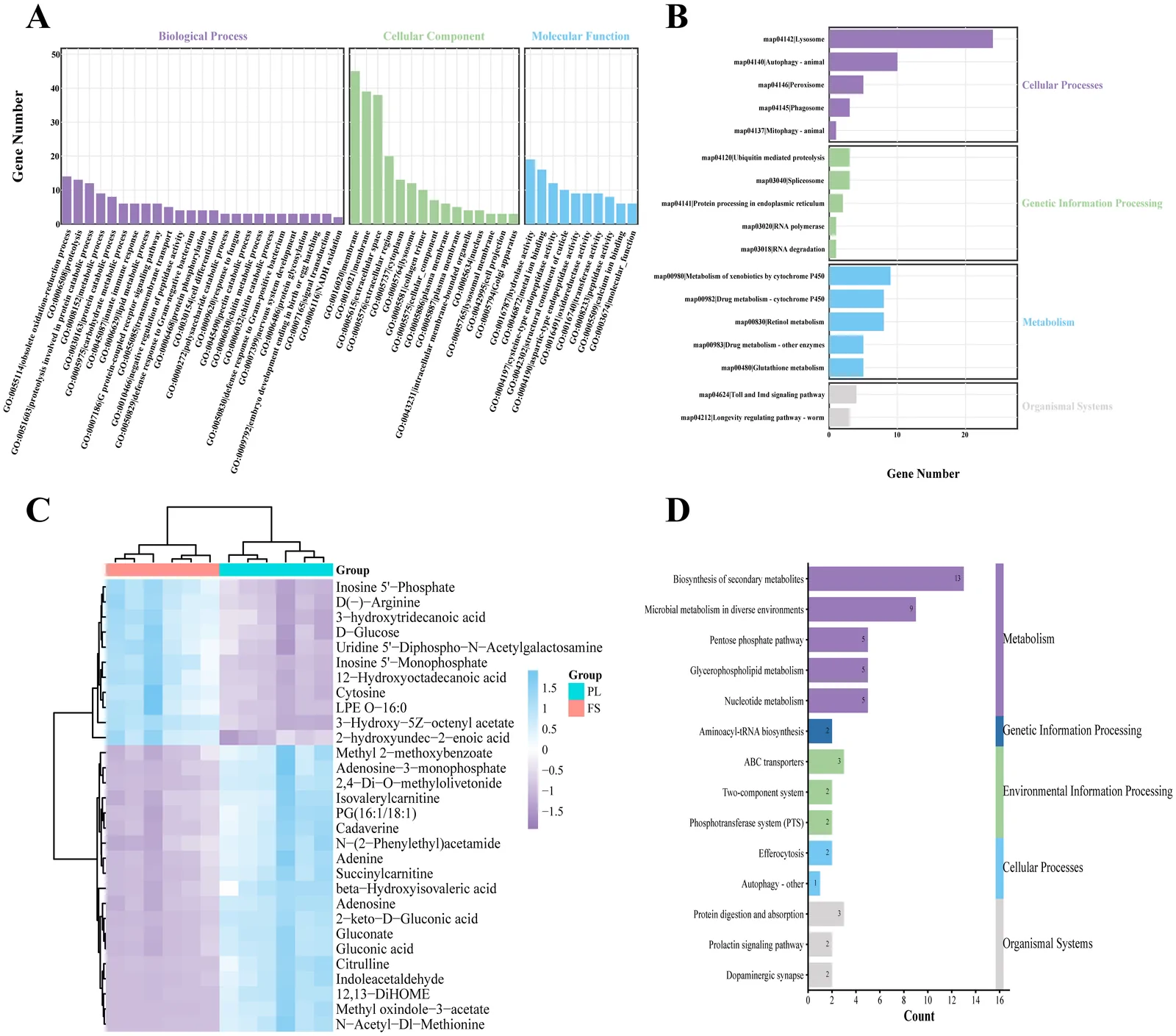
The analysis of sensitivity differences between PL and FS strains using transcriptomics and metabolomics. **(A)** GO classification of DEGs screened from transcriptomic analysis (n = 4). **(B)** KEGG pathway functional enrichment for DEGs (n = 4). **(C)** The expression patterns of the top 30 DAMs in heatmap (n = 6). **(D)** KEGG pathway enrichment analysis of DAMs (n = 6).

Validation of the orthogonal partial least squares discriminant analysis (OPLS-DA) model was performed using 200 permutation tests. The resulting intercepts for R² and Q² were 0.69 and –0.72, respectively, demonstrating satisfactory model stability and reproducibility, thus supporting subsequent qualitative and quantitative assays (**Figures 1F, G**). Key metabolites of glycolysis and the tricarboxylic acid (TCA) cycle accounted for the majority in the top 30 DAMs (**Figure 2C**). KEGG enrichment highlighted biosynthesis of secondary metabolites, microbial metabolism in diverse environments and pentose phosphate pathway (**Figure 2D**).

For further transcriptomics analysis, we identified five major pathways including ascorbate and aldarate metabolism (degree = 16), glycolysis/gluconeogenesis (15), fatty acid degradation (14), drug metabolism - cytochrome P450 (12), and metabolism of xenobiotics by cytochrome P450 (12, **Figure 3A**). Integrating the above pathways, we found that the glycolysis/gluconeogenesis and pentose phosphate pathway exhibited strong correlation and functional complementarity.

**Figure 3:**
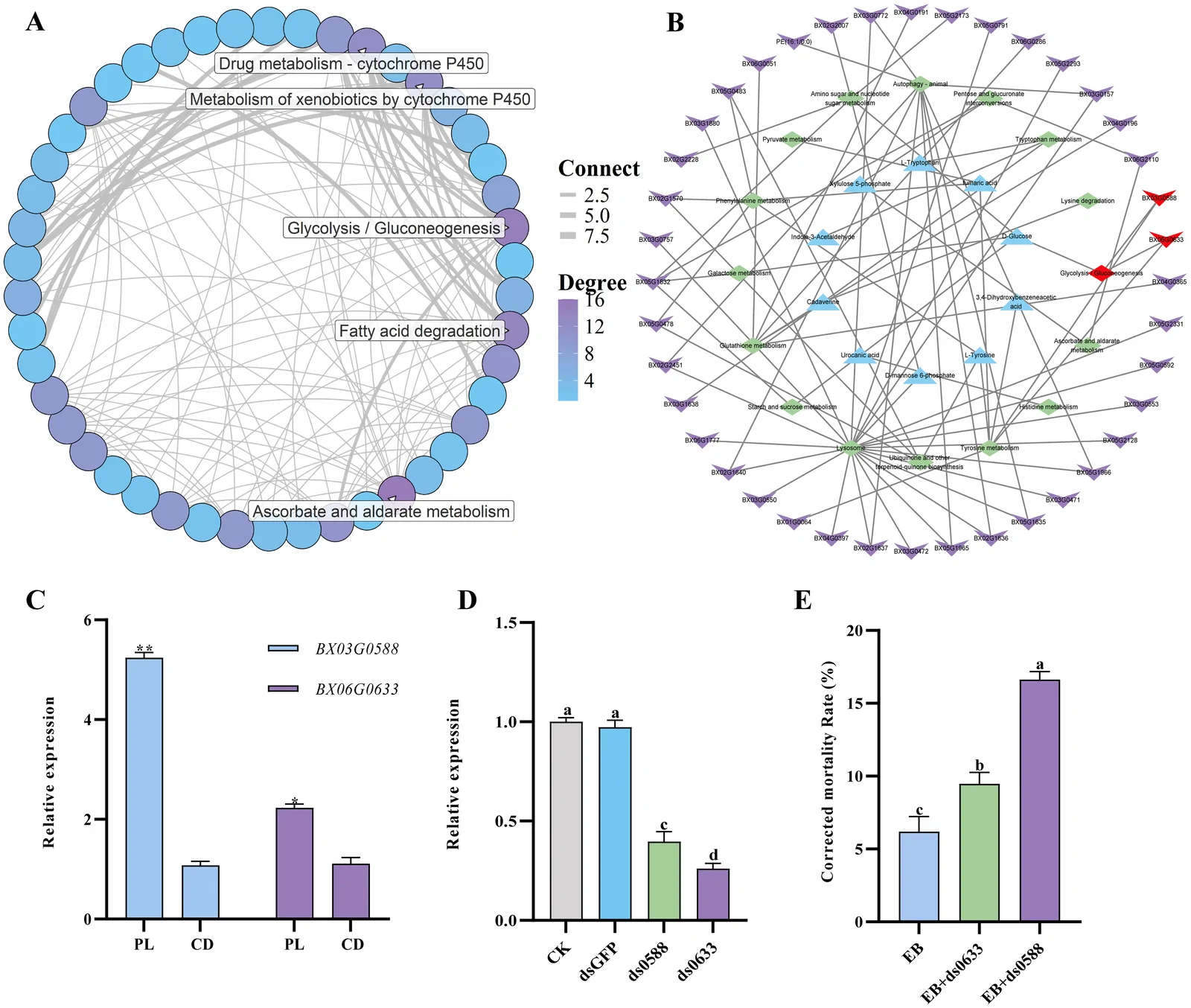
Screening potential pathway and gene associated with sensitivity differences in PL and FS trains using multi-omics integrated analysis. **(A)** Transcriptomic analysis identified the top five enriched pathways. **(B)** Network plot from transcriptomic-metabolomic integrated analysis. Red represents the key pathway and genes. **(C)** Relative expression of *BX03G0588* and *BX06G0633* in PL and FS strains after emamectin benzoateon treatment (n=3) **(D)** RNAi silencing efficiency of *BX03G0588* and *BX06G0633* in PL strains (n=3). **(E)** Corrected mortality rate following emamectin benzoateon alone and combined treatments of dsRNA with emamectin benzoateon (n=3). Differences between the two groups were analyzed using t-test. * *p* < 0.05 and ** *p* < 0.01. Different letters indicate statistically significant differences based on Duncan’s multiple range test (*p* < 0.05).

We performed integrated transcriptome and metabolome analyses. The results revealed strong positive correlations between multiple pathways and metabolites, suggesting that alterations in metabolite accumulation may be directly or indirectly regulated by the corresponding genes. A total of 17 pathways were enriched. Combined with transcriptome data, we ultimately selected the glycolysis/gluconeogenesis pathway as a potential regulatory pathway for further investigation. Notably, only two genes (*BX03G0588* and *BX06G0633*) were annotated to this pathway, and both exhibited significant correlations with D-Glucose (**Figure 3B**).

### 3.4 Effects of dsRNA on the sensitivity of *B. xylophilus* to EB

*BX03G0588* and *BX06G0633* in PL strain were confirmed to be up-regulated by RT-qPCR, compared with CD strain (**Figure 3C**). Compared to the sterile water group, gene silencing efficiencies of ds0588 and ds0633 in PL strain were 60.33% and 74.67%, respectively (**Figure 3D**). Compared with EB treatment, the corrected mortality of mixture treatments increased by 3.28% and 10.44% for ds0588 and ds0633, respectively (**Figure 3E**). Meanwhile, the LC_50_ value after ds0588+EB treatment was 162.56 μg•mL^-1^ (**Table 4**).

## 4 Discussion

Human activities are accelerating the biological evolution of natural populations. The increased frequency and dosage of nematicide application impose continuous selective pressure on nematodes, driving their rapid evolution (Poulin et al., 2026). In our study, the sensitivity of 15 *B. xylophilus* strains from different regions to EB was evaluated. The result of RR < 5 showed that strains from Shandong Province may not have developed resistance. However, the current criteria for sensitivity evaluation were adopted from arthropods. Thus, the present results are only for reference, we will establish a complete set of standard procedures specifically for *B. xylophilus* to meet the practical evaluation requirements.

Nematodes are widely distributed in nature. Given their species richness, parasitic nematodes have caused severe damage to agricultural and livestock production, including intestinal parasitic nematodes in animals and soil nematodes. For instance, gastrointestinal nematodes in cattle and *Haemonchus contortus* had developed severe resistance to macrocyclic lactones, with resistance levels increasing annually (Gasbarre, 2014; Poulin et al., 2026; Luo et al., 2017). Similarly, the LC_50_ values of *Meloidogyne incognita* to osthiazate and fluopyram were 5.4 and 2.3 mg•L^-^¹, respectively, which were significantly higher than those of sensitive populations (Fujikawa et al., 2025). We hypothesize that the temporary absence of resistance in *B. xylophilus* is related to its living traits and environment. A prerequisite for resistance development is continuous external selection pressure. However, the primary strategy against *B. xylophilus* is trunk injection, which presents two issues. On the one hand, commercial formulations vary in adjuvants across different regions, affecting the water solubility and systemic translocation of EB within pine trees, thereby reducing nematode exposure to the active ingredient (Gworek et al., 2021; Li et al., 2019; Liu et al., 2023). On the other hand, considering the stability of the active ingredient, current management practices do not meet the requirements for precise application, resulting in the short persistence of EB within trees and insufficient doses to maintain the health of pine trees (Li and Fantke, 2022; Hazra and Purkait, 2019; Huang et al., 2021; Matthies and Beulke, 2017). Regarding the biochemical parameters of the PL and CD strains, the detoxification enzyme activities in the PL strain were substantially higher than those in the CD strain, with significant differences observed in SOD and GST activities. This pattern is typical in the evolution and indicates that field populations of *B. xylophilus* are under selection pressure from nematicides (Cai et al., 2021; Khan et al., 2020; Della Vechia et al., 2021).

To explore the basis for this different sensitivity, we integrated multi-omics analyses. Transcriptomics revealed that the significant enrichment pathways were associated with cytochrome P450 (CYP450), glycolysis/gluconeogenesis, and fatty acid degradation pathways. However, the CYP450 pathway responds broadly to exogenous stress, making it difficult to identify specific responsive genes (Nauen et al., 2022; Lu et al., 2021; Ye et al., 2022). Therefore, combining transcriptomics and metabolomics analyses, we identified the glycolysis/gluconeogenesis pathway and validated the expression changes of *BX03G0588* and *BX06G0633* in this pathway using RT-qPCR. Glycolysis converts glucose into pyruvate and provides substantial energy for cellular processes. Insecticide-resistant insects showed significant upregulation of glycolysis-related genes to increase ATP production, supplying energy needed for detoxification and counteracting pesticide-induced oxidative damage, which was consistent with the result of lower MDA level in the PL strain (Zhao et al., 2023). To verify the relationship between candidate genes and nematode sensitivity, we performed RNAi and reassessed the EB toxicity to PL strain. The results demonstrated that *BX03G0588* was associated with sensitivity. Many reports showed that RNAi treatment restored pest sensitivity to pesticides and enabled effective management against pest. These findings highlighted not only the RNAi as a validation tool but also the *BX03G0588* as a potential target for *B. xylophilus* control (Li et al., 2023; Christiaens et al., 2020).

Nevertheless, we acknowledge several limitations in our study. This is only a snapshot and cannot rule out the future development of resistance. In the future, the number of field-collected samples and the collected period should be increased to enable continuous surveillance of resistance development in *B. xylophilus*. Meanwhile, the effects of symbiotic bacteria inside nematodes on nematicide metabolism were not taken into account in our study. We only preliminarily evaluated the sensitivity of nematodes after direct exposure to EB. In follow-up research, we will identify the stable symbiotic bacterial species carried by nematodes to comprehensively explore the causes of differential sensitivity among different strains. And, this study lacks validation of direct physiological indicators linking nematode sensitivity to the glycolysis/gluconeogenesis pathway, and the current results are merely based on multi-omics conjoint analysis. In subsequent research, we will further explore this pathway and conduct comprehensive functional verification of *BX03G0588*. Moreover, to mitigate the risk of resistance resulting from the prolonged use of a single nematicide, alternative compounds should be explored to reduce the application rate of EB. These efforts will contribute to the improvement of sustainable management strategies for PWD while ensuring biosafety.

## 5 Conclusion

The toxicity of EB against 15 *B. xylophilus* strains from different regions revealed that maximum and minimum LC_50_ values were 320.99 and 114.63 μg•mL^-1^, respectively, yielding a resistance ratio of 2.8. These results indicated that *B. xylophilus* strains in Shandong Province may not have developed resistance to EB. Following EB treatment, the PL strain exhibited significantly lower MDA levels and higher activities of detoxifying enzymes than the CD strain. Integrated multi-omics analysis and RNAi treatments suggested that this difference in sensitivity may be associated with the gene *BX03G0588* in the glycolysis/gluconeogenesis pathway of the PL strain.

## Data Availability

The raw transcriptome sequencing data have been deposited in the Genome Sequence Archive (GSA), accession number PRJCA075172.

## References

[B1] Akami A. Fukuda K. (2025). Relationship between the distribution of the pinewood nematode (Bursaphelenchus xylophilus) and the development of xylem embolism in the stems of Japanese black pine (Pinus thunbergii) seedlings monitored by magnetic resonance imaging. Phytopathology 115, 172–180. doi: 10.1094/phyto-06-24-0191-r 39373566

[B2] Cai H. Yang L. Zuo Z. Liao W. Yang Z. (2021). Resistance status of Myzus persicae to pesticide and its relationship with enzymes. Agron. J. 113, 806–819. doi: 10.1002/agj2.2049041531421

[B3] Cha D. J. Kim D. S. Lee S. K. Han H. R. (2019). A new on-site detection method for Bursaphelenchus xylophilus in infected pine trees. For. Pathol. 49, e12503. doi: 10.1111/efp.1250340046247

[B4] Christiaens O. Niu J. Taning C. N. T. (2020). RNAi in insects: a revolution in fundamental research and pest control applications. Insects 11, 415. doi: 10.3390/insects11070415 32635402 PMC7411770

[B5] Delgado-Rodriguez M. Rodríguez-Soalleiro R. Nobre S. R. Rodriguez L. C. E. Diaz-Balteiro L. (2025). Resin: not only a secondary forest product. Eur. J. For. Res. 144, 1161–1169. doi: 10.1007/s10342-025-01800-630311153

[B6] Della Vechia J. F. Van Leeuwen T. Rossi G. D. Andrade D. J. (2021). The role of detoxification enzymes in the susceptibility of Brevipalpus californicus exposed to acaricide and insecticide mixtures. Pestic. Biochem. Physiol. 175, 104855. doi: 10.1016/j.pestbp.2021.104855 33993973

[B7] Feng J. Li R. Wang C. Yang H. Deng W. Du G. . (2022). Nematicidal phytochemicals against pine wood nematode, Bursaphelenchus xylophilus (nematoda: aphelenchoididae). J. Plant Dis. Prot. 130, 215–223. doi: 10.1007/s41348-022-00692-730311153

[B8] Fujikawa A. Perry R. N. Toyota K. (2025). Occurrence of a population of the root-knot nematode, Meloidogyne incognita, with low sensitivity to two major nematicides, fosthiazate and fluopyram, in Japan. Pest Manage. Sci. 81, 698–706. doi: 10.1002/ps.8468 39373621 PMC11716370

[B9] Gao Y. Yoon K. A. Lee J. H. Kim J. H. Lee S. H. (2022). Overexpression of glutamate-gated chloride channel in the integument is mainly responsible for emamectin benzoate resistance in the western flower thrips Frankliniella occidentalis. Pest Manage. Sci. 78, 4140–4150. doi: 10.1002/ps.7032 35686450

[B10] Gasbarre L. C. (2014). Anthelmintic resistance in cattle nematodes in the US. Vet. Parasitol. 204, 3–11. doi: 10.1016/j.vetpar.2014.03.017 24798800

[B11] Gupta A. Ghosh P. Arora K. Sharma S. Kumar S. (2023). Valorization potential of pine needle waste biomass: recent trends and future perspectives. Environ. Sci. pollut. Res. 31, 36136–36151. doi: 10.1007/s11356-023-27440-0 37166735

[B12] Gworek B. Kijeńska M. Wrzosek J. Graniewska M. (2021). Pharmaceuticals in the soil and plant environment: a review. Water Air Soil pollut. 232, 145. doi: 10.1007/s11270-020-04954-830311153

[B13] Hazra D. K. Purkait A. (2019). Role of pesticide formulations for sustainable crop protection and environment management: a review. J. Pharmacogn. Phytochem. 8, 686–693.

[B14] Huang G. Deng Y. Zhang Y. Feng P. Xu C. Fu L. . (2021). Study on long-term pest control and stability of double-layer pesticide carrier in indoor and outdoor environment. Chem. Eng. J. 403, 126342. doi: 10.1016/j.cej.2020.12634238826717

[B15] Huang X. Li Q. Chen J. Liu W. Guo K. Hu J. . (2024). Molecular characterization and functional analysis of genes mediating emamectin benzoate action to the pinewood nematode (Bursaphelenchus xylophilus). Pestic. Biochem. Physiol. 205, 106148. doi: 10.1016/j.pestbp.2024.106148 39477601

[B16] Khan M. M. Kaleem-Ullah R. M. Siddiqui J. A. Ali S. (2020). Insecticide resistance and detoxification enzymes activity in Nilaparvata lugens Stål against neonicotinoids. J. Agric. Sci. 12, 24. doi: 10.5539/jas.v12n5p24

[B17] Koo C.-D. Lee H.-Y. Han J.-H. Sung J.-H. Shin J.-H. (2013). Infection behavior and distribution of Bursaphelenchus xylophilus in Pinus densiflora trees. For. Sci. Technol. 9, 81–86. doi: 10.1080/21580103.2013.80116837339054

[B18] Lai M. Dong L. Su R. Zhang L. Jia T. Chen T. . (2023). Needle functional features in contrasting yield phenotypes of slash pine at three locations in southern China. Ind. Crops Prod. 206, 117613. doi: 10.1016/j.indcrop.2023.11761338826717

[B19] Lee J.-W. Mwamula A. O. Choi J.-H. Lee H.-W. Kim Y. S. Kim J.-H. . (2023). The potency of abamectin formulations against the pine wood nematode, Bursaphelenchus xylophilus. Plant Pathol. J. 39, 290–302. doi: 10.5423/ppj.oa.02.2023.0023 37291769 PMC10265120

[B20] Lee S.-C. Lee H.-R. Kim D.-S. Kwon J.-H. Huh M.-J. Park I.-K. (2020). Emamectin benzoate 9.7% SL as a new formulation for a trunk-injections against pine wood nematode, Bursaphelenchus xylophilus. J. For. Res. 31, 1399–1403. doi: 10.1007/s11676-018-00873-x30311153

[B21] Li Z. Fantke P. (2022). Considering degradation kinetics of pesticides in plant uptake models: proof of concept for potato. Pest Manage. Sci. 79, 1154–1163. doi: 10.1002/ps.7288 36371622 PMC10099551

[B22] Li Y. Sallach J. B. Zhang W. Boyd S. A. Li H. (2019). Insight into the distribution of pharmaceuticals in soil-water-plant systems. Water Res. 152, 38–46. doi: 10.1016/j.watres.2018.12.039 30660096

[B23] Li M. Wang M. Yang T. Xu M. Li Y. Pei Y. . (2024). Optimized emamectin benzoate trunk injection: addressing temperature limitations for pine wilt disease control. Pest. Manage. Sci. 81, 892–902. doi: 10.1002/ps.8493 39425489

[B24] Li B.-J. Wang K.-K. Yu Y. Wei J.-Q. Zhu J. Wang J.-L. . (2023). PxRdl2 dsRNA increased the insecticidal activities of GABAR-targeting compounds against Plutella xylostella. Pestic. Biochem. Physiol. 195, 105548. doi: 10.1016/j.pestbp.2023.105548 37666591

[B25] Liang W. Yu A. Wang G. Zheng F. Jia J. Xu H. (2018). Chitosan-based nanoparticles of avermectin to control pine wood nematodes. Int. J. Biol. Macromol. 112, 258–263. doi: 10.1016/j.ijbiomac.2018.01.174 29382581

[B26] Lin N. Su X. Zhou X. Zhou L. Chen A. Hu J. . (2024). Characteristics and conductivity of emamectin benzoate-inclusive nanocapsule in Pinus massoniana Lamb. Forests 15, 444. doi: 10.3390/f1503044430654563

[B27] Liu J. Cheng J. Zhou C. Ma L. Chen X. Li Y. . (2023). Uptake kinetics and subcellular distribution of three classes of typical pesticides in rice plants. Sci. Total Environ. 858, 159826. doi: 10.1016/j.scitotenv.2022.159826 36374729

[B28] Lu F. Guo K. Chen A. Chen S. Lin H. Zhou X. (2020). Transcriptomic profiling of effects of emamectin benzoate on the pine wood nematode Bursaphelenchus xylophilus. Pest. Manage. Sci. 76, 747–757. doi: 10.1002/ps.5575 31386282

[B29] Lu K. Song Y. Zeng R. (2021). The role of cytochrome P450-mediated detoxification in insect adaptation to xenobiotics. Curr. Opin. Insect Sci. 43, 103–107. doi: 10.1016/j.cois.2020.11.004 33387688

[B30] Luo X. Shi X. Yuan C. Ai M. Ge C. Hu M. . (2017). Genome-wide SNP analysis using 2b-RAD sequencing identifies the candidate genes putatively associated with resistance to ivermectin in Haemonchus contortus. Parasitol. Vectors 10, 586. doi: 10.1186/s13071-016-1959-6 28095895 PMC5240194

[B31] Ma T. Wang Q. Wei P. Zhu K. Feng A. He Y. . (2022). EGCG-gelatin biofilm improved the protein degradation, flavor and micromolecule metabolites of tilapia fillets during chilled storage. Food Chem. 375, 131662. doi: 10.1016/j.foodchem.2021.131662 34865925

[B32] Matthies M. Beulke S. (2017). Considerations of temperature in the context of the persistence classification in the EU. Environ. Sci. Eur. 29, 1. doi: 10.1186/s12302-017-0113-1 28424754 PMC5380697

[B33] Mitrea-Calin M. Inacio M. L. Cean M. Costache C. Rusinque L. Paraschiv M. . (2020). First detection of Bursaphelenchus mucronatus (Nematoda: Aphelenchoididae) on Monochamus sutor (Coleoptera: Cerambycidae) in Romania. For. Pathol. 50, e12578. doi: 10.1111/efp.1257840046247

[B34] Mo R. Wang Y. Dong S. Ma J. Mo Y. (2023). Ecosystem service evaluation and multi-objective management of Pinus massoniana Lamb. plantations in Guangxi, China. Forests 14, 213. doi: 10.3390/f1402021330654563

[B35] Nauen R. Bass C. Feyereisen R. Vontas J. (2022). The role of cytochrome P450s in insect toxicology and resistance. Annu. Rev. Entomol. 67, 105–124. doi: 10.1146/annurev-ento-070621-061328 34590892

[B36] Nian B. Ye J.-R. Wu X.-Q. (2026). Cloning and functional analysis of glutathione S-transferase gene BxGST3 and BxGST1 in Bursaphelenchus xylophilus. Front. Plant Sci. 17, 1847982. doi: 10.3389/fpls.2026.1847982 42267124 PMC13243375

[B37] Pan L. Wang D. Chen A. Zhang S. Jia J. Wang J. . (2018). Early diagnosis of plant disease via NIR spectroscopy: a study in Bursaphelenchus xylophilus disease. Int. J. Robot. Autom. 33, 321–326. doi: 10.2316/Journal.206.2018.3.206-5535

[B38] Poulin R. Bennett J. Blasco-Costa I. Dutra D. D. A. Doherty J.-F. Dowle E. . (2026). Scientists' warning on the rapid evolution of parasites in the Anthropocene. Evol. Appl. 19, e70244. doi: 10.1111/eva.70244 42057856 PMC13122726

[B39] Susaeta A. Gutiérrez E. Lozano S. (2023). Profit-efficiency analysis of forest ecosystem services in the southeastern US. Ecosyst. Serv. 64, 101567. doi: 10.1016/j.ecoser.2023.10156738826717

[B40] Teng F. Wang Z. Xue M. Li H. Wen Q. Xu P. . (2025). Development of metal-organic framework-based emamectin benzoate nanopesticides for effective control of Bursaphelenchus xylophilus. Langmuir 41, 7455–7466. doi: 10.1021/acs.langmuir.4c04975 40062902

[B41] Wang X. Song P. An B. Smagghe G. Han S. Feng S. . (2026). Laboratory selection reveals flubendiamide resistance, fitness costs, and detoxification-related mechanisms in Spodoptera frugiperda: insights for insecticide resistance management. Pestic. Biochem. Physiol. 219, 107019. doi: 10.1016/j.pestbp.2026.107019 41831887

[B42] Xu H. Luo Y. Zhang T. Shi Y. (2011). Changes of reflectance spectra of pine needles in different stage after being infected by pine wood nematode. Spectrosc. Spectral Anal. 31, 1352–1356. doi: 10.3964/j.issn.1000-0593(2011)05-1352-05 21800599

[B43] Ye M. Nayak B. Xiong L. Xie C. Dong Y. You M. . (2022). The role of insect cytochrome P450s in mediating insecticide resistance. Agriculture 12, 53. doi: 10.3390/agriculture1201005330654563

[B44] Zeng Q. Li L. Yao Y. Fang B. Shi Y. Yang Y. . (2026). The F116V mutation and overexpression of CYP9A186 confer high-level resistance to emamectin benzoate and abamectin in Spodoptera exigua. Pestic. Biochem. Physiol. 218, 106969. doi: 10.1016/j.pestbp.2026.106969 41629036

[B45] Zhang W. Ni A. Zhang J. Sun G. Xiang F. Cheng H. . (2026). Residual dynamics of fluopyram and its compound formulations in Pinus massoniana and their efficacy in preventing pine wilt disease. Plants 15, 302. doi: 10.3390/plants15020302 41600109 PMC12845286

[B46] Zhang W. Wu X. Ye J. Li C. Hu L. Lin R. . (2023). Toxicity of an emamectin benzoate microemulsion against Bursaphelenchus xylophilus and its effect on the prevention of pine wilt disease. Forests 14, 1476. doi: 10.3390/f1407147630654563

[B47] Zhao J. Huang J. Yan J. Fang G. (2020). Economic loss of pine wood nematode disease in mainland China from 1998 to 2017. Forests 11, 1042. doi: 10.3390/f1110104230654563

[B48] Zhao Y. Zou C. Zhang L. Li C. Li X. Song L. (2023). Chlorbenzuron caused growth arrest through interference of glycolysis and energy metabolism in Hyphantria cunea (Lepidoptera: Erebidae) larvae. Pestic. Biochem. Physiol. 193, 105466. doi: 10.1016/j.pestbp.2023.105466 37248002

